# Metal-Free Ligand-Centered Radical Scavenging in Pyridine-Modified Tetraaza Macrocycles

**DOI:** 10.1002/ejoc.70635

**Published:** 2026-06-29

**Authors:** Christina Mantsorov, Hannah K. Pyle, Tahmina Afroz, Caden-Jack N. Walls, Nora Del Bosque, Timothy M. Schwartz, Chelsea N. Miller, Kristof Pota, Benjamin G. Janesko, Kayla N. Green

**Affiliations:** Department of Chemistry and Biochemistry, Texas Christian University, Fort Worth, Texas, USA

**Keywords:** chemistry, delocalized electron, density functional theory, DPPH, free radical scavenger, ligand, pyridinophane, radical, redox, substituent

## Abstract

Pyridine-modified tetraaza macrocycles are widely studied as ligand platforms for metal coordination, yet their intrinsic redox and radical reactivity in the absence of metals remains unclear. Here, we report the unexpected finding that pyridinophane Py_2_N_2_ and PyN_3_ macrocycles act as efficient metal-free radical scavengers toward the stable free radical 2,2-diphenyl-1-picrylhydrazyl (DPPH•). Systematic evaluation of substituent effects reveals distinct structure–reactivity relationships. PyN_3_ derivatives exhibit a linear dependence on electronic parameters, consistent with a donor-controlled regime, whereas Py_2_N_2_ macrocycles show a pronounced parabolic dependence on resonance and inductive effects, indicative of cooperative stabilization. ^1^H NMR spectroscopy supports the formation of a transient ligand–DPPH• encounter complex, followed by a rapid hydrogen-atom transfer (HAT). Density functional theory identifies the β-C–H position on the macrocyclic backbone as the thermodynamically preferred hydrogen-donor site. The resulting ligand-centered radical is formed with the Py_2_N_2_ and stabilized through delocalization across the macrocyclic framework, enabling a captodative-like electronic environment that is inaccessible to the PyN_3_ scaffold. Together, these findings establish a ligand-centered HAT mechanism governed by macrocyclic topology rather than metal coordination and highlight a previously unrecognized design principle for electronically tunable, metal-free macrocyclic radical scavengers.

## 1 | Introduction

Reactive oxygen species (ROS), such as superoxide (O_2_•^−^), hydroxyl radical (•OH), and hydrogen peroxide (H_2_O_2_), are continuously generated in aerobic organisms as by-products of normal metabolism [1–3]. While tightly regulated ROS levels serve essential roles in cellular signaling, unrestrained accumulation induces oxidative stress, leading to DNA damage, lipid peroxidation, and protein oxidation [4–6]. Cells rely on both enzymatic (e.g., superoxide dismutase, catalase, peroxidases) and nonenzymatic (e.g., glutathione, ascorbate, tocopherol) defense systems to maintain redox homeostasis [7–10]. Synthetic molecules capable of quenching reactive radicals or decomposing peroxides are therefore valuable tools for probing oxidative processes and mitigating redox imbalance in biological and materials contexts [11, 12].

At the molecular level, most small-molecule antioxidants act through hydrogen-atom transfer (HAT) or single-electron transfer (SET) pathways that neutralize reactive species to form thermodynamically stable, nonpropagating radicals [13–15]. In HAT mechanisms, two parameters drive the overall efficiency: the bond dissociation energy (BDE) of the hydrogen-donor bond and the stability of the resulting radical [16–19]. Lower BDEs and greater radical delocalization generally enhance antioxidant reactivity [16, 20, 21]. Phenolic antioxidants, such as BHT (butylated hydroxytoluene) and Trolox, illustrate this principle. The phenoxyl radicals generated upon H-atom abstraction are resonance stabilized, yielding high scavenging rates toward peroxyl and alkoxyl radicals [22–24]. These relationships form the conceptual foundation for structure–activity correlations in antioxidant chemistry.

Small-molecule antioxidants, such as BHT, Trolox, TEMPO, and ascorbic acid, are widely used as benchmarks in radical scavenging assays, including DPPH•, although their reported activities are highly dependent on experimental conditions [25–28]. In this context, the development of alternative antioxidant scaffolds that enable systematic tuning of reactivity remains of interest. Macrocyclic systems offer a potentially advantageous platform, where structural rigidity and electronic modulation can be leveraged to probe radical stabilization mechanisms.

**FIGURE 1 |:**
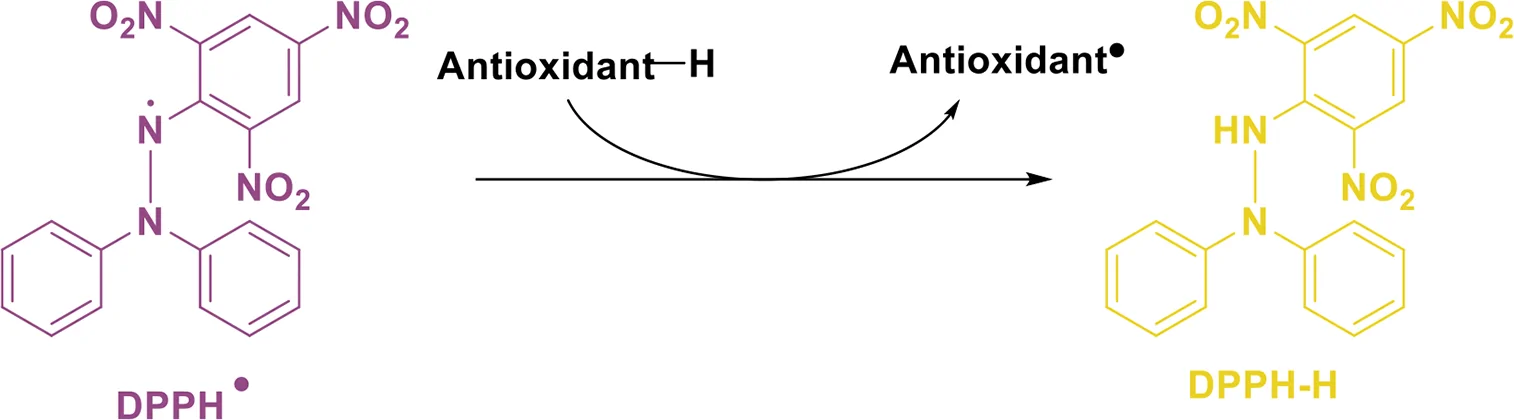
Schematic illustration of the reduction of the DPPH radical by an antioxidant to give DPPH-H, showing the accompanying color change from deep violet to pale yellow. Revised from references [29, 30].

The DPPH• assay (2,2-diphenyl-1-picrylhydrazyl, Figure 1) provides a robust and reproducible means of probing such reactivity. The deep violet DPPH• radical (*λ*_max_ = 517 nm) is reduced to DPPH-H via HAT or SET, allowing quantitative evaluation of radical scavenging capacity [29, 30]. The assay specifically reflects intrinsic bond strength and radical stabilization rather than nonspecific oxidation because the DPPH• radical is sterically hindered and reacts only with sufficiently strong H-atom donors [31, 32]. Substituent effects on the DPPH• radical quenching are often analyzed using classical Hammett- or Swain–Lupton-type linear free-energy relationships (LFERs), in which inductive (*σ*_*i*_) and resonance (*σ*^R^) parameters quantify how substituents modulate electronic structure and reactivity [33–35].

The captodative effect (the simultaneous stabilization of a radical by adjacent electron-donating (+R) and electron-withdrawing (−I) substituents) provides a powerful framework for understanding substituent synergy [36–38]. Originally formulated to rationalize the exceptional persistence of α-amino and α-alkoxy radicals, the captodative concept has since been used to understand radical stabilization in carbon-centered species, aminooxyl radicals, and conjugated donor–acceptor systems [38–40]. The effect arises because the donor disperses spin density, while the acceptor lowers the energy of the singly occupied orbital, collectively reducing the radical’s overall reactivity. Despite its broad theoretical support, experimental evidence of captodative stabilization within macrocyclic or heteroaromatic frameworks remains rare [41, 42].

Macrocyclic ligands provide an appealing platform to explore such phenomena because their rigid geometries and preorganized donor arrays enable delocalization of unpaired spin density across multiple atoms, potentially stabilizing ligand-centered radicals even in the absence of metal ions [43, 44]. For example, cyclambased ligands have been shown to undergo ligand-centered oxidation under certain conditions, while redox-active salen and porphyrinoid systems display intraligand electron transfer during oxidative catalysis [45–48]. Yet direct examination of metal-free radical scavenging activity in macrocycles remains uncommon, leaving the fundamental electronic factors governing this reactivity largely uncharacterized.

**FIGURE 2 |:**
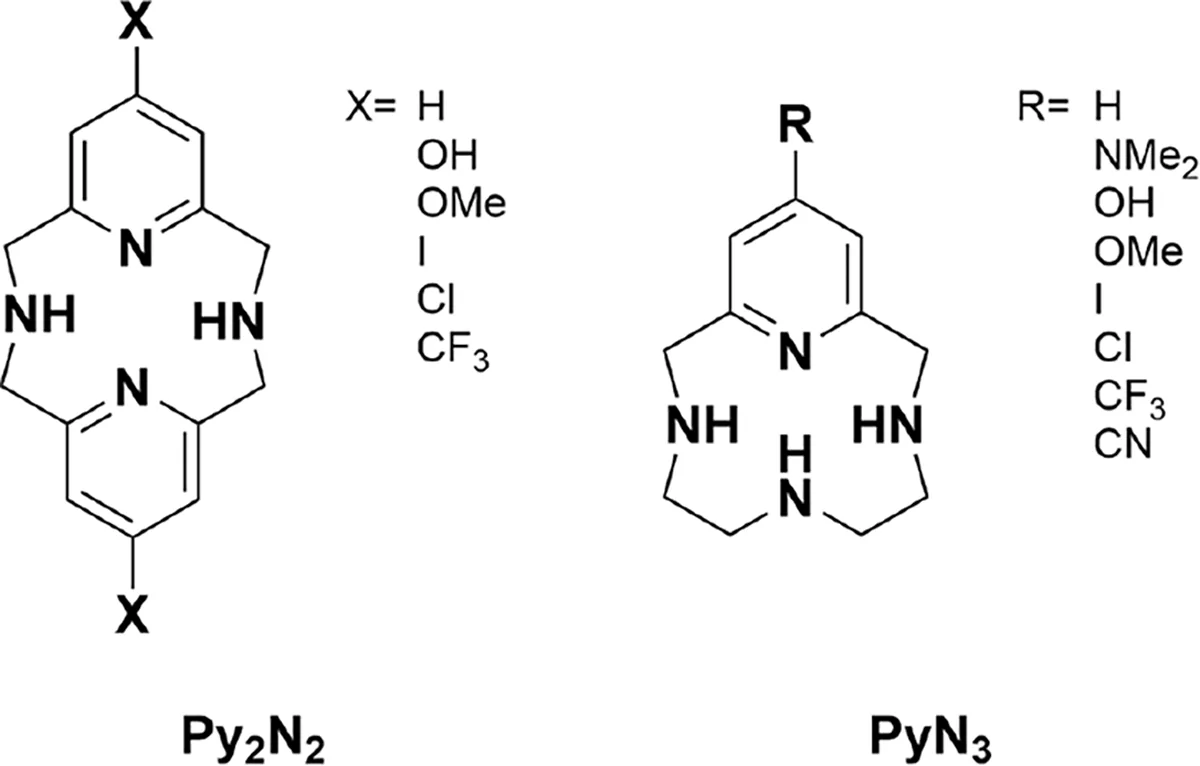
Ligands assessed in this article, previously reported in the literature [49–51].

Our group and others have developed pyridine-modified tetraaza macrocycles (PyN_3_ and Py_2_N_2_; Figure 2) as modular scaffolds for redox catalysis and mitigation of oxidative stress [49, 50, 52]. These ligands, which combine polyamine macrocyclic frameworks with conjugated *para*-substituted pyridyl units, were designed for metal coordination in catalytic or biomimetic ROS transformations. During control experiments, however, we observed a surprising result. The free ligands *sans* metal ions were capable of quenching the DPPH• radical. This exciting observation prompted a mechanistic investigation of their intrinsic redox reactivity and the electronic factors that enable this activity.

All ligands examined herein are previously reported and characterized molecules [49–51]. The present study focuses on new mechanistic and electronic structure observations, revealing an unanticipated metal-independent antioxidant pathway in these established macrocyclic systems. Beyond its immediate implications for antioxidant design, this discovery provides a rare experimental example of captodative radical stabilization within a heteroaromatic macrocycle, expanding our understanding of how ligand topology and electronic effects govern radical reactivity.

## 2 | Results and Discussion

### 2.1 | Radical Scavenging Profiles

The radical scavenging capacity of each pyridinophane (Figure 2) was assessed using the DPPH• assay in triplicate [53–57]. The *para*-substituted Py_2_N_2_ and PyN_3_ families both displayed clear concentration-dependent quenching of the DPPH• absorption band (*λ* = 517 nm), indicating that the ligands are competent hydrogen atom or electron donors.

**FIGURE 3 |:**
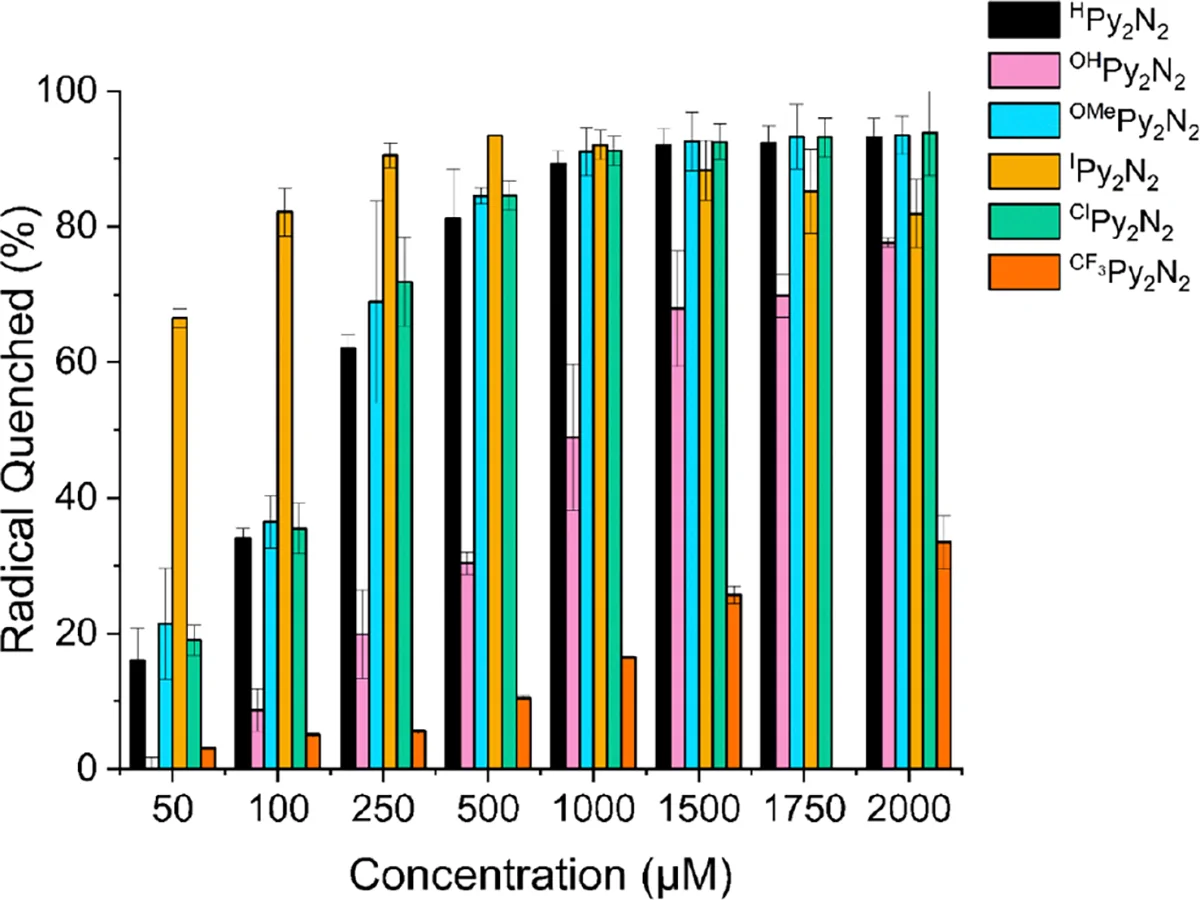
Concentration-dependent radical scavenging profiles for the Py_2_N_2_ ligand series in methanol, expressed as the percent DPPH• quenching at each tested concentration. Error bars represent the standard deviation of three independent measurements.

For the Py_2_N_2_ series (Figure 3), quenching efficiency increased steeply with ligand concentration and approached completion for most derivatives between 250 and 1000 μM. The unsubstituted (H-Py_2_N_2_) and methoxy-substituted (OMe-Py_2_N_2_) analogues quenched around 85%–90% of the DPPH• radical at 500 μM, whereas the halogenated members (I- and Cl-Py_2_N_2_) were even more potent, achieving a greater than 90% quench at only 250 μM. Direct SET from the halogen substituents themselves is unlikely, as aryl halides are inductively electron withdrawing and are not known to function as efficient electron donors toward DPPH•. The enhanced activity of the halogenated Py_2_N_2_ derivatives is, therefore, more consistent with substituent-mediated stabilization of a ligand-centered radical following HAT.

In contrast, the strongly electron-withdrawing CF_3_-Py_2_N_2_ exhibited the least activity (33% at 2000 μM). The hydroxy derivative (OH-Py_2_N_2_) demonstrated intermediate efficiency (70% quench at 2000 μM), likely reflecting competing intramolecular hydrogen bonding and possible keto–enol tautomerization, both of which can limit H-atom donation [51, 58].

The resulting order of activity, I ≈ Cl > OMe ≈ H > OH ≫ CF_3_, suggests an optimum when both mild resonance donation and moderate inductive withdrawal are present, consistent with a captodative-like stabilization of the ligand-centered radical intermediate. Notably, the rapid decay of the DPPH• absorbance for I- and Cl-Py_2_N_2_ implies a kinetically facile HAT pathway rather than a stepwise electron transfer (ET) mechanism, consistent with the near-instantaneous color loss observed experimentally. Compared to BHT, which we have previously shown to quench 100% of the DPPH• at concentrations greater than 250 μM, these ligands would be considered slightly weaker radical scavenging agents [51].

**FIGURE 4 |:**
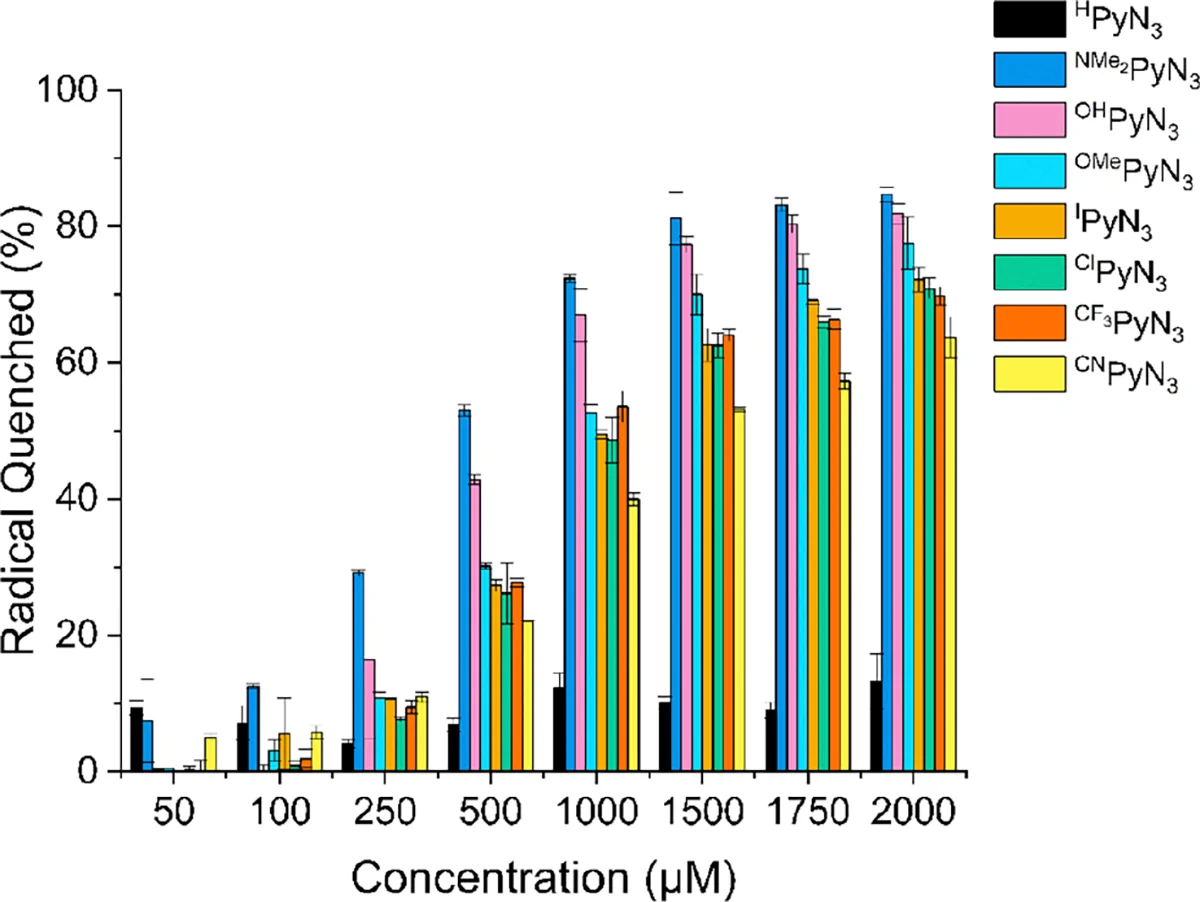
Concentration-dependent radical scavenging profiles for the PyN_3_ ligand series in methanol, expressed as percent DPPH• quenching at each tested concentration. Error bars represent the standard deviation of three independent measurements.

The PyN_3_ series (Figure 4) exhibited weaker, slower DPPH• quenching. Electron-donating substituents (NMe_2_, OMe, and OH) afforded the most active species, reaching 75%–85% quench only at mM concentrations, whereas halogenated and electron-poor ligands (I-, Cl-, CF_3_-, and CN-PyN_3_) were considerably less effective. The corresponding trend, NMe_2_ ≈ OMe ≈ OH > I ≈ Cl > CF_3_ ≈ CN ≫ H, indicates that radical scavenging within this single-pyridine framework depends primarily on resonance donation, which lowers the BDE involved in HAT. Notably, the unsubstituted H-PyN_3_ analog exhibits the lowest activity, consistent with the absence of any substituent-derived radical stabilization in a scaffold where delocalization is already limited to a single aryl unit. The markedly higher efficiency of the Py_2_N_2_ series compared with PyN_3_ highlights the role of the second pyridine unit, which provides additional pathways for delocalization of the unpaired electron formed after HAT. This effect does not arise from one ring acting purely as a donor and the other as an acceptor; rather, the two aryl units together create an electronic environment that better stabilizes the radical intermediate. The observed activity sequence also qualitatively follows the relative BDE trends predicted from substituent electronic parameters, implying that both *σ*^R^ and *σ*_*i*_ contributions modulate the ease of H-atom donation.

These findings position the Py_2_N_2_ scaffold among a growing class of metal-free macrocyclic antioxidants, in which macrocyclic rigidity and extended conjugation stabilize organic radicals. In contrast to classical antioxidants such as BHT, Trolox, or TEMPO, typically evaluated by DPPH• quenching with reported IC_50_ values spanning the low micromolar to tens of μg mL^−1^ range depending on assay conditions [25–28], the present system offers a distinct advantage in that reactivity is electronically tunable through systematic substituent variation. While direct comparison across studies is complicated by differences in solvent, DPPH concentration, and reaction conditions, this tunability enables mechanistic interrogation of radical stabilization within a consistent experimental framework. This behavior is explored further through analysis of substituent effects using LFERs.

### 2.2 | Linear Free-Energy Relationships

**FIGURE 5 |:**
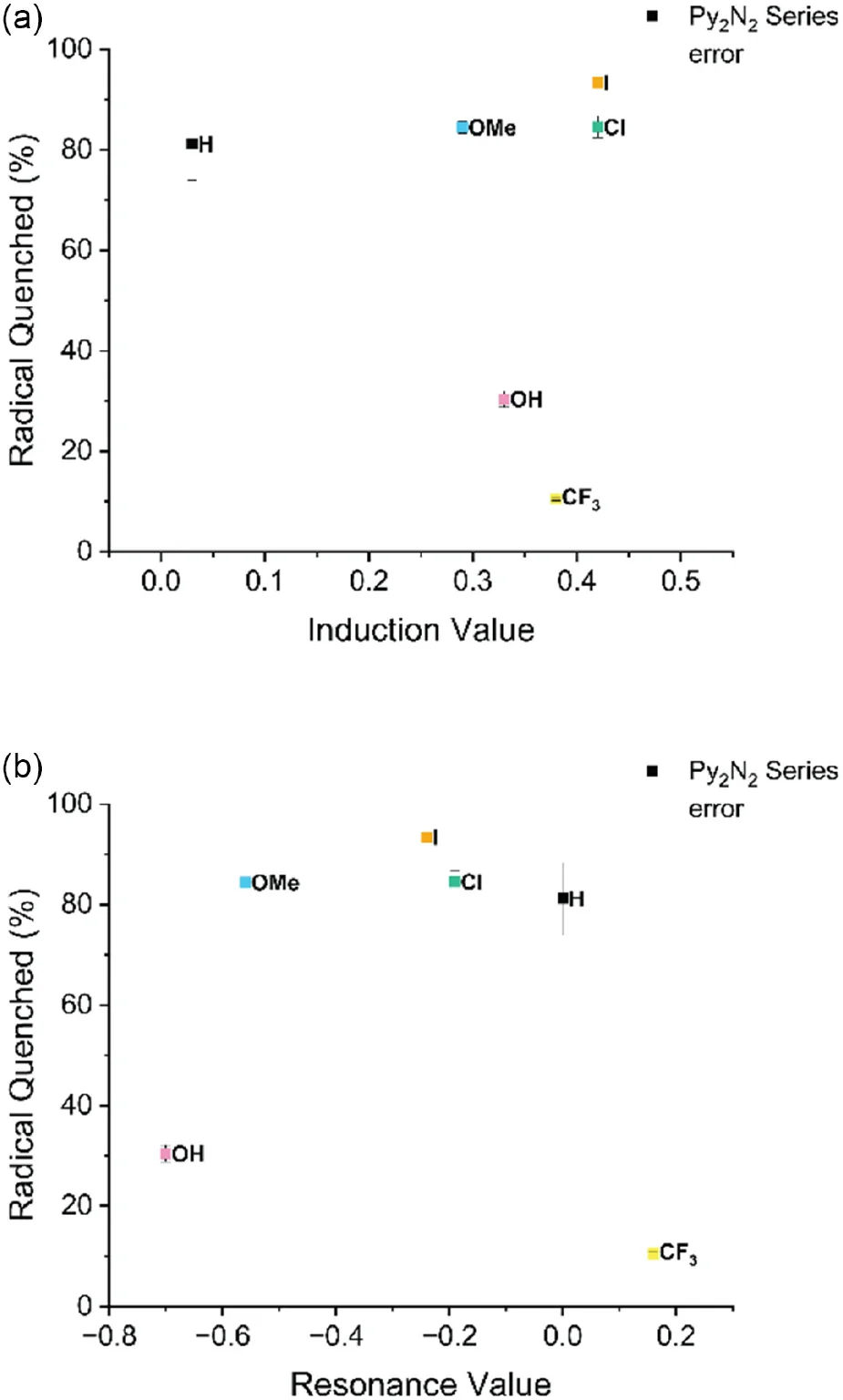
LFERs for the Py_2_N_2_ ligand families at 500 μM in methanol: (a) percent DPPH• radical quenched versus inductive (*σ*_*i*_) substituent parameters; (b) percent DPPH• radical quenched versus resonance (*σ*^R^) parameters.

To quantify how substituent electronics influence metal-free radical scavenging, the percentage of DPPH• radical quenched was correlated with the Swain–Lupton inductive (*σ*_*i*_) and resonance (*σ*^R^) parameters (Figure 5) for each molecule at 500 μM [59]. These dual-parameter LFERs provide a quantitative framework for dissecting how +R and −I effects modulate the X–H BDE and the stability of the ligand-centered radical formed during HAT. Comparable approaches have been used to interpret substituent effects in phenolic and aminic antioxidants, where similar trends correlate with BDE lowering and enhanced radical delocalization [33, 60, 61].

For the Py_2_N_2_ series (Figure 5a), radical scavenging efficiency shows a shallow parabolic or saturating profile as *σ*_*i*_ increases. Activity rises from 81% for H-Py_2_N_2_ (*σ*_*i*_ = 0.03) to 84% for OMe (*σ*_*i*_ = 0.29), then reaches its maximum at I-Py_2_N_2_ (93%, *σ*_*i*_ = 0.42), with Cl-Py_2_N_2_ also high (84%, *σ*_*i*_ = 0.42). Efficiency drops for strong electron-withdrawing functionalizations, falling to 10% for CF_3_-Py_2_N_2_ (*σ*_*i*_ = 0.38). OH-Py_2_N_2_ (*σ*_*i*_ = 0.33) is unusually weak (30%), consistent with intramolecular H-bonding that impedes H-atom donation. The resulting curvature reflects a captodative-like balance. Modest −I stabilizes the developing radical and lowers the effective donor BDE, whereas excessive −I depletes electron density and slows HAT. Thus, halogenated Py_2_N_2_ ligands (I > Cl) define the optimum region where donor and acceptor effects cooperate.

**FIGURE 6 |:**
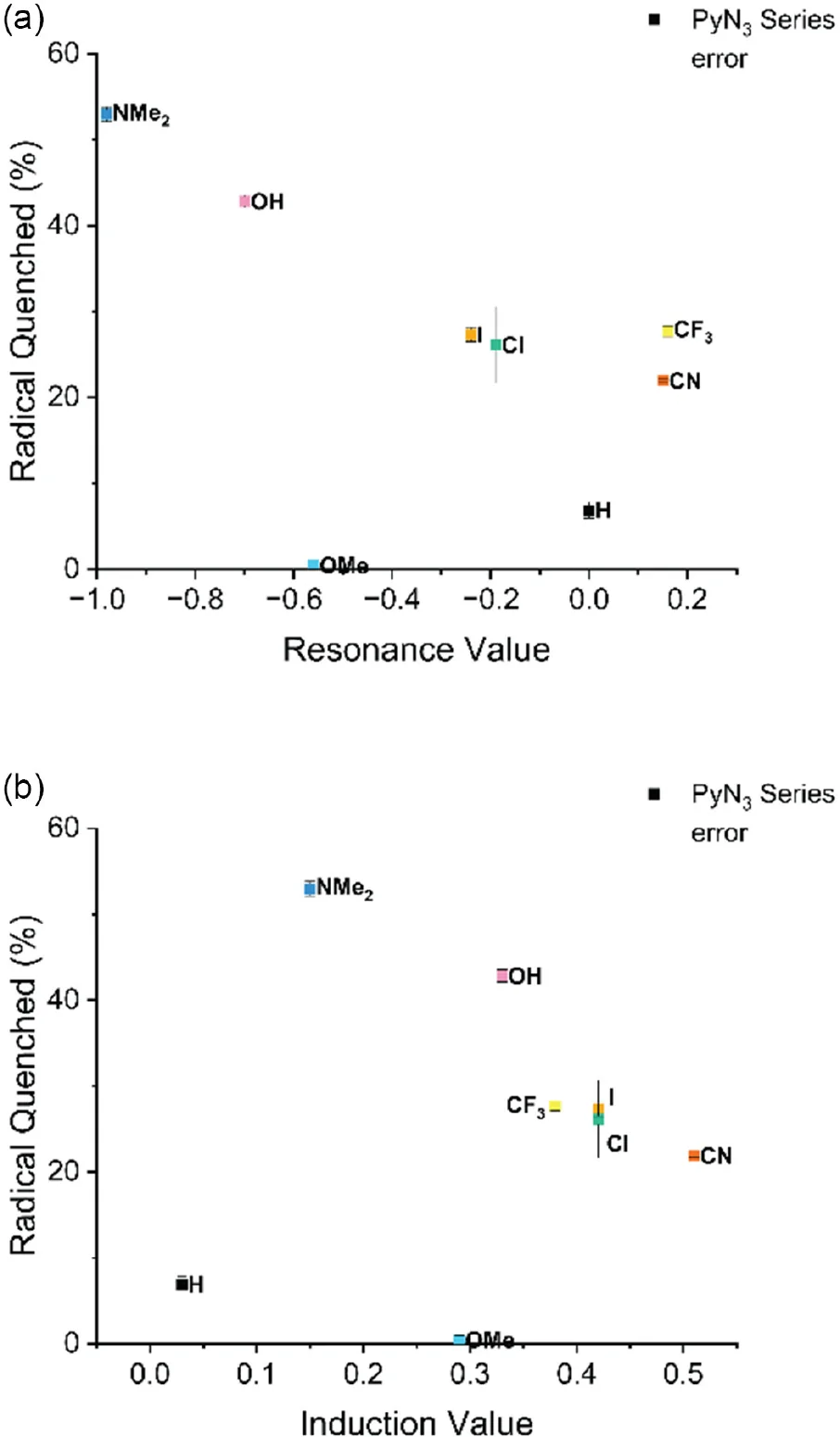
LFERs for the PyN_3_ ligand families at 500 μM in methanol: (a) percent DPPH• radical quenched versus inductive (*σ*_*i*_) substituent parameters; (b) percent DPPH• radical quenched versus resonance (*σ*^R^) parameters.

In contrast, the PyN_3_ series displays a strictly linear inverse correlation between *σ*_*i*_ and percentage of DPPH• radical quenched (Figure 6a). Activity is highest for NMe_2_-PyN_3_ (53%, *σ*_*i*_ = 0.15) and declines monotonically for more withdrawing substituents (e.g., CF_3_, Cl, I, CN: 22–28%). No curvature or optimum is observed, indicating that inductive withdrawal uniformly suppresses reactivity in this single-pyridine topology. This contrast highlights the structural role of the second pyridine unit in the Py_2_N_2_ scaffold. Incorporation of a second heteroaromatic ring expands the electronic framework available for radical stabilization, enabling broader delocalization of spin density and more effective coupling between substituent effects and the reactive center. As a result, Py_2_N_2_ ligands access a cooperative electronic regime in which resonance and inductive effects can act synergistically, giving rise to the observed parabolic behavior that is absent in the single-pyridine PyN_3_ system.

For Py_2_N_2_ (Figure 5b) pyridinophanes, the percentage of DPPH quenched versus *σ*^R^ exhibits a clear parabolic dependence. Activity increases from H-Py_2_N_2_ (*σ*^R^ = 0.00, 81%) to a maximum across intermediate donors: I-Py_2_N_2_ (93%, *σ*^R^ = −0.24) and OMe- and Cl-Py_2_N_2_ (both 84%, *σ*^R^ = −0.56 and −0.19, respectively). Efficiency falls for strong donors (NMe_2_, *σ*^R^ = −0.98) and those with withdrawing character (CF_3_, *σ*^R^ = +0.16; 10%). The optimum, centered around *σ*^R^ = −0.4 to −0.2, indicates that limited +R lowers the donor BDE while concurrent modest −I disperses spin density through the macrocycle, an experimental manifestation of captodative-like stabilization in a heteroaromatic macrocyclic framework. The OH-Py_2_N_2_ outlier (*σ*^R^ = −0.70, 30%) is consistent with H-bonding that counteracts any nominal +R benefit.

In contrast, PyN_3_ follows a monotonic donor-controlled trend where activity increases with more negative *σ*^R^: from 7% for H-PyN_3_ (*σ*^R^ = 0.00) to 53% for NMe_2_-PyN_3_ (*σ*^R^ = −0.98), with all other substituents between 20% and 45%. The absence of curvature confirms that HAT in PyN_3_ is driven solely by resonance-induced bond weakening, with no dual donor–acceptor stabilization.

These quantitative relationships validate that electronic substituent parameters (*σ*_*i*_, *σ*^R^) can predict metal-free radical scavenging trends in macrocyclic systems, to provide a rational basis for the design of next-generation tunable antioxidants. The mechanistic implications of this electronic control were further investigated through NMR spectroscopic experiments.

### 2.3 | Mechanistic Insight From NMR Studies

**FIGURE 7 |:**
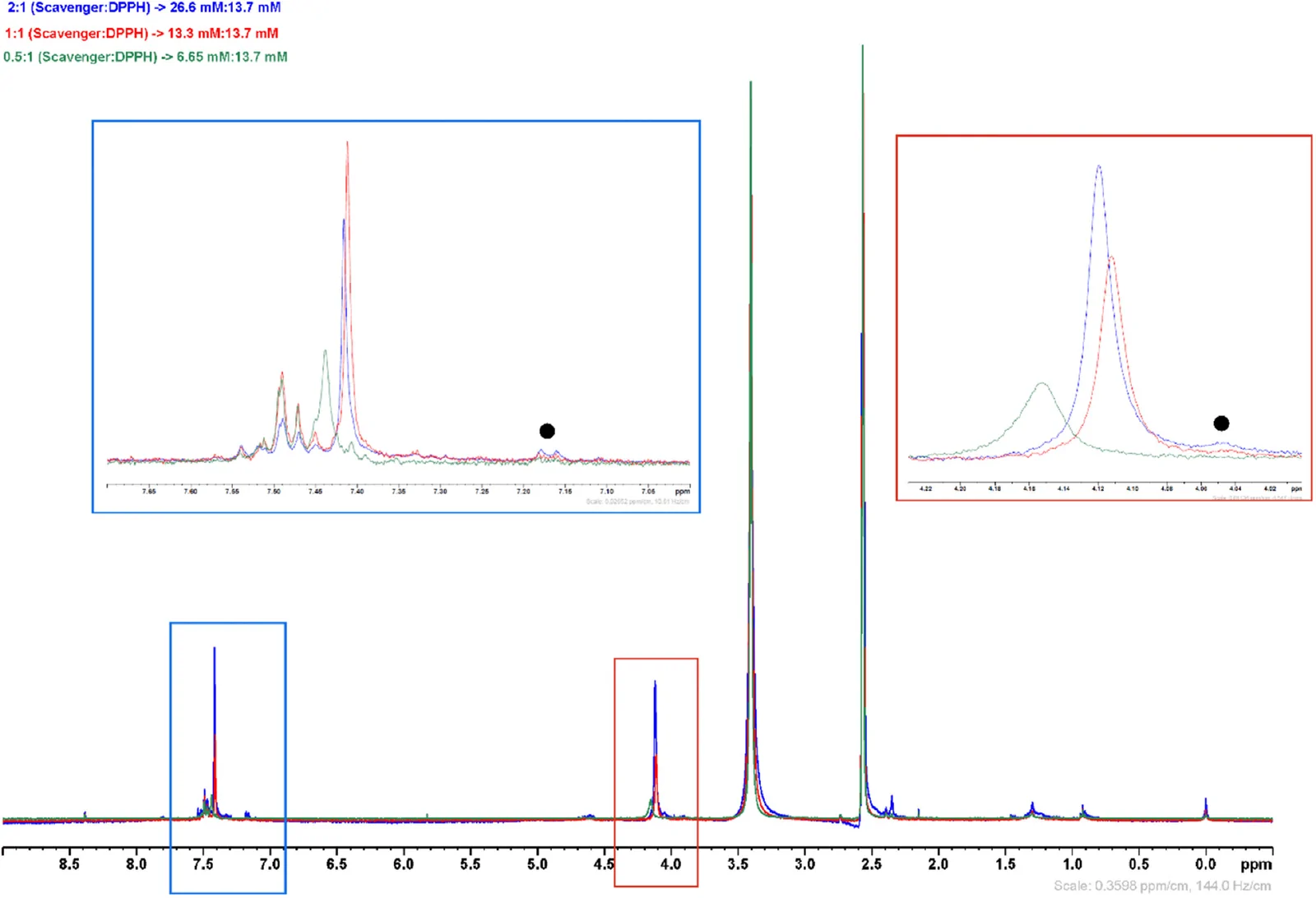
^1^H NMR spectra of I-Py_2_N_2_ with DPPH at ligand/DPPH molar ratios of 2:1 (blue), 1:1 (red), and 0.5:1 (green). Spectra were acquired in DMSO-d_6_ following 15 min of equilibration under ambient light. Insets highlight selected aromatic and aliphatic regions showing concentration-dependent line broadening, chemical shift changes, and the emergence of minor resonances (black circles).

To elucidate the mechanism of DPPH• radical quenching and determine whether HAT or ET pathways predominate, the interaction between I-Py_2_N_2_ and the DPPH• radical was examined by ^1^H NMR spectroscopy in DMSO-d_6_. Spectra were recorded after 15 min exposure to ambient light at ligand-to-radical ratios of 0.5:1, 1:1, and 2:1 (Figure 7).

At 0.5:1, the aromatic resonances of I-Py_2_N_2_ were shifted downfield by 0.5 ppm, became markedly broadened, and their intensities dropped by more than expected for simple dilution. Such extensive broadening arises from paramagnetic relaxation caused by rapid exchange between the ligand and excess DPPH• radical, which indicates the formation of a weak outersphere encounter complex in which unpaired spin density is transiently shared [62–64]. The behavior is characteristic of fast HAT exchange or *π*-stacking/halogen-assisted preassociation, rather than slow ET.

At 1:1, line broadening persisted but decreased, and the resonances partially recovered. When the ratio was increased to 2:1, the ligand peaks sharpened further and shifted slightly upfield, while new minor signals appeared that were absent at lower ratios. These features coincide with attenuation of the resonances derived from the DPPH• radical, consistent with partial radical consumption and the formation of a new diamagnetic product mixture. The appearance of new resonances without extensive chemical shift dispersion suggests the formation of ligand-derived species, most plausibly the reduced DPPH–H product and a transient ligand-centered radical formed via HAT.

Following addition of 16 μL D_2_O directly to the previously acquired DMSO-d_6_ sample, all I-Py_2_N_2_ peaks shifted modestly downfield, confirming the involvement of exchangeable protons. At 0.5:1 and 1:1, the magnitude of these shifts was uniform across the spectrum, whereas at 2:1 the newly emergent minor peaks exhibited stronger downfield motion and decreased intensity. DPPH-derived signals were more perturbed under these conditions, inferring that the products at high ligand excess contain hydrogen-bonded or protonated species (e.g., DPPH-H and/or H-bonded I-Py_2_N_2_•H). The modest intensity increase of an aromatic resonance near the proposed radical site after D_2_O addition suggests a decrease in exchange rates or altered local hydrogen bonding at that position.

The collective pattern, progressive broadening, sharpening, and appearance of new peaks with increasing [I-Py_2_N_2_], accompanied by downfield D_2_O shifts, parallel the reaction progress observed in the UV–Vis DPPH assay. The results are consistent with (i) the formation of a weak, paramagnetically broadened encounter complex between DPPH• and I-Py_2_N_2_; (ii) rapid HAT to generate DPPH-H; and (iii) transient formation of a ligand-centered radical whose spin density is delocalized across the macrocyclic framework and pyridyl units. The absence of persistent new paramagnetic features suggests fast exchange or subsequent relaxation rather than accumulation of a stable radical species.

These NMR results reinforce the mechanistic picture derived from the LFER analysis. The high activity of I- and Cl-Py_2_N_2_ corresponds to an optimal combination of mild inductive withdrawal and limited resonance donation, which stabilizes the nascent radical through captodative balance. Within the Py_2_N_2_ scaffold, this push–pull electronic environment lowers the effective X–H BDE and distributes spin density, enabling rapid yet controlled HAT reactivity. The observed trends are consistent with captodative stabilization reported for aminyl and α-amino radicals, in which substituents providing inductive withdrawal, often including halogens, cooperate with resonance donation elsewhere in the framework to maximize radical persistence [65–67].

Collectively, the ^1^H NMR titrations provide direct spectroscopic evidence that the DPPH• radical quenching by Py_2_N_2_ macrocycles proceeds via a metal-independent, ligand-centered HAT mechanism stabilized by captodative electronic delocalization, consistent with the electronic structure trends established in Sections 2.1 and 2.2.

### 2.4 | Computational Studies

To corroborate the HAT reactivity observed experimentally in PyN_3_ and Py_2_N_2_, density functional theory (DFT) analysis was performed to provide theoretical quantification of both BDEs and relative stabilities of radicals produced. For this study, the homolytic fission pathway was initially considered. Several functional and basis set combinations were screened; the M06–2X/def2-TZVP was ultimately selected for BDE computational analysis due to its lowest (most stable) computed energies. Geometries were optimized both in gas phase and solution using SMD (water).

**FIGURE 8 |:**
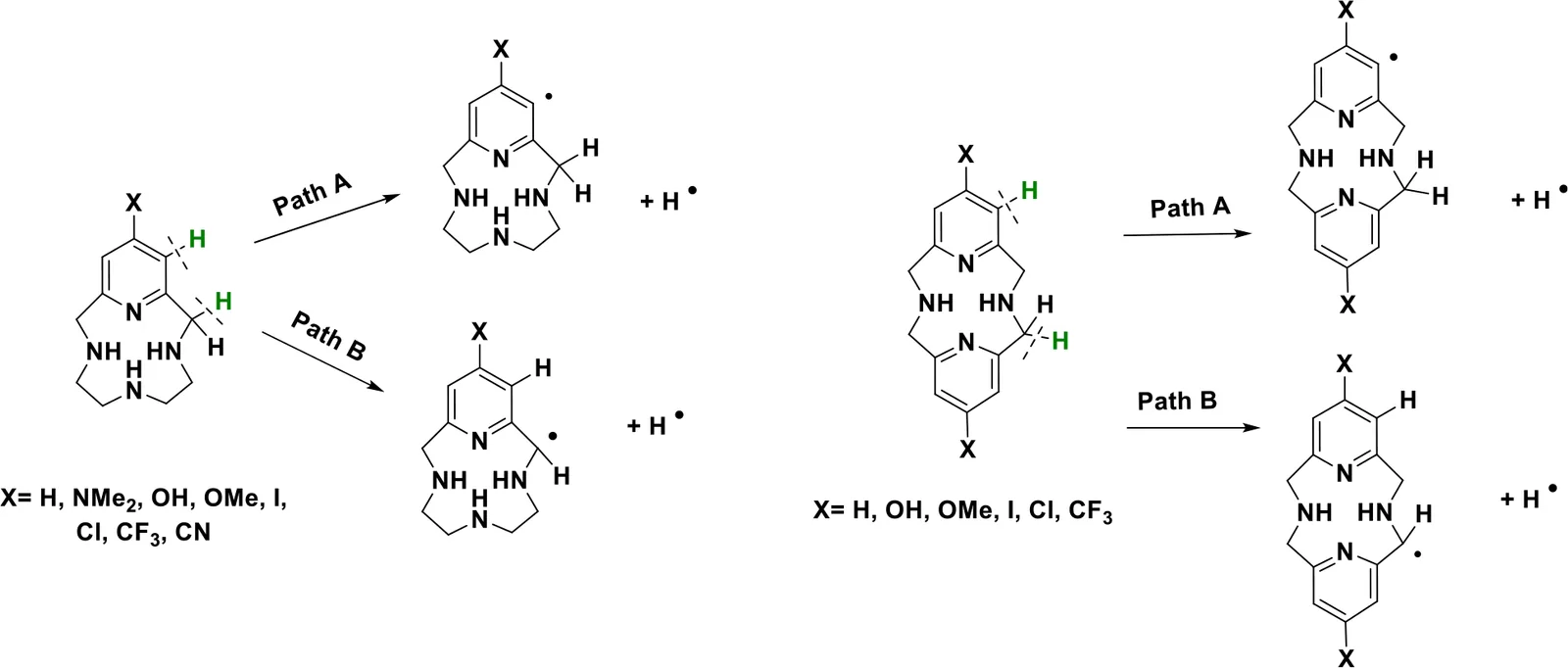
PyN_3_ and Py_2_N_2_ ligands showing the two C–H sites examined by DFT: pyridine C–H (Path A) and macrocyclic β-C–H adjacent to the pyridine substituent (Path B).

Two potential reactive sites, the pyridine C–H bond at the 3-position (Path A) and the β-C–H bond on the macrocyclic ring adjacent to the pyridine substituent (Path B), shown in Figure 8, were analyzed based on the ability for the captodative effect to occur.

Across all Py_2_N_2_ compounds studied (Table 1), Pathway B is consistently more favorable than Pathway A in both gas phase and SMD water solvation. Radicals from Pathway A are higher in energy by 32–36 kcal/mol in gas phase and 28–31 kcal/mol in water, confirming a strong thermodynamic preference for Pathway B. Solvation effects further highlight this difference. Pathway B energies increase by 6–7 kcal/mol, whereas Pathway A shows only a modest increase of 2–3 kcal/mol, indicating greater solvent sensitivity for radicals formed via Pathway B.

Substituent tuning is minimal, with the lowest energies observed for CF_3_-Py_2_N_2_ and the highest for OMe-Py_2_N_2_. However, the overall range for Pathway B in water is very small only ≈1.9 kcal/mol, suggesting that selectivity is governed more likely by the core scaffold rather than substituent effects. Mechanistically, Pathway A radicals reside on unsaturated carbons within the pyridine ring, stabilized by resonance. In contrast, Pathway B radicals occur on saturated carbons adjacent to multiple nitrogen, enabling strong hyperconjugation and inductive stabilization. These combined electronic and structural features fundamentally drive the persistent preference for Pathway B across all environments and substituent variations.

The increased dispersion of unpaired radicals from Pathway B in more stable configurations reduces subsequent reactivity (with DPPH). The increased stability of these radicals results in weaker C–H bond and lower BDE, both key drivers for radical scavenging reactivity.

Similar to the observations made in the Py_2_N_2_ series, Pathway B explored with PyN_3_ molecules resulted in lower ΔBDE values (Table 2) when compared to Pathway A in both gas phase and the SMD (water). Additionally, a similar thermodynamic preference for Pathway B over Pathway A is observed, with Pathway B producing lower average energies of 32 kcal/mol in gas phase, as well as average lower energies of 19–20 kcal/mol in the solvated model. As expected, the solvated model produced higher energies in both pathways for PyN_3_ molecules from interactions with resulting radicals and water molecules. Overall, Pathway B can be seen to be the preferred method of HAT radical generation based on computational methods in the PyN_3_ series.

Differences between the Py_2_N_2_ and PyN_3_ macrocycles can be observed with comparison of the energies of parent molecules to the respective substituents. Energies of Pathway A for Py_2_N_2_ and PyN_3_ in both gas phase and water model are 1–2 kcal/mol within each other. In contrast, Pathway B affords higher energy values for PyN_3_ when compared to Py_2_N_2_ by an average 4–5 kcal/mol in gas phase, and 12 kcal/mol in the solvated model. Most notably, the ΔBDE of Cl-Py_2_N_2_ compared to Cl-PyN_3_ in Pathway B showed a remarkable difference of 37–38 kcal/mol. These observations suggest that there is an overall better stability observed through both Pathway A and Pathway B in the Py_2_N_2_ parent molecules when compared to the PyN_3_ parent molecules. Moreover, unlike the Py_2_N_2_ molecules, the substituents play a much larger role in stabilizing the resultant radical species of the PyN_3_ molecules.

### 2.5 | Proposed Mechanistic Framework

**FIGURE 9 |:**
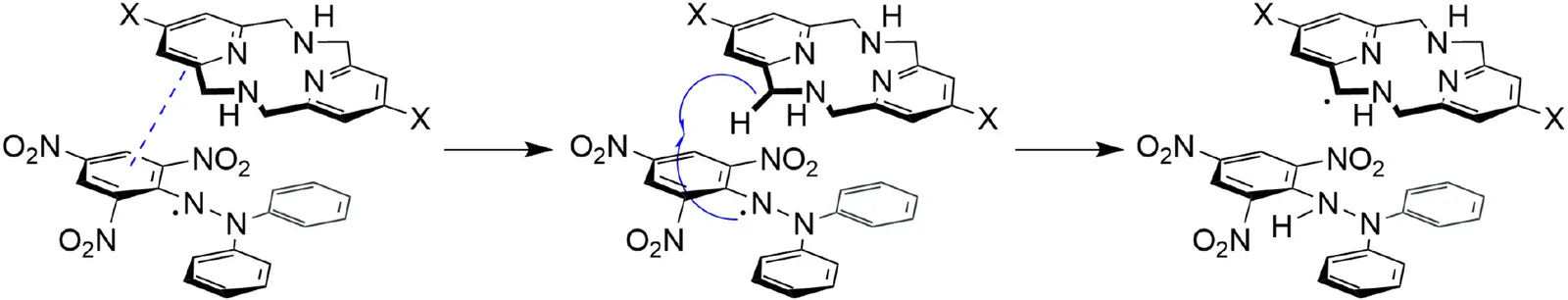
Proposed mechanistic pathway for the radical scavenging ability of the Py_2_N_2_ series.

The combined experimental and computational data support a ligand-centered HAT mechanism for the DPPH• radical quenching by pyridine-modified tetraaza macrocycles. In this process, radical scavenging proceeds without metal coordination and is instead governed by macrocyclic topology and substituent-dependent electronic modulation (Figure 9).

The reaction is initiated by the formation of a weak outersphere encounter complex between the DPPH• radical and the macrocycle, likely stabilized by *π*–*π* interactions between aromatic surfaces and, in the case of halogenated ligands, additional dispersive or halogen–*π* contacts. Evidence for such preassociation is provided by pronounced line broadening and chemical shift perturbations observed in the ^1^H NMR spectra of I-Py_2_N_2_ at substoichiometric ligand/DPPH• ratios, consistent with rapid exchange of spin density between the paramagnetic radical and the diamagnetic ligand.

The chemically productive step is proposed to be a concerted HAT from the macrocycle to the DPPH• radical, yielding DPPH–H and a transient ligand-centered radical. DFT calculations successfully identify the β-C–H position of the macrocyclic backbone as the preferred donor site, with homolytic cleavage at this position strongly favored over pyridyl C–H abstraction across all ligands examined. Although calculated BDEs vary only modestly with substitution, the computational results establish that the macrocyclic scaffold itself is intrinsically capable of supporting HAT, while experimental substituent effects arise primarily from kinetic and electronic modulation rather than large thermodynamic differences in bond strength.

The efficiency of HAT is dictated by how effectively the resulting ligand-centered radical is stabilized. In the Py_2_N_2_ series, abstraction at the β-C–H position generates a radical adjacent to both an amine donor and an aryl substituent. Although the two pyridine units are not directly conjugated to the same carbon center, the bis-aryl framework modifies the overall electronic environment of the macrocycle, allowing greater delocalization and inductive tuning compared to the mono-aryl PyN_3_ system. Linear free-energy analyses reveal a parabolic dependence of radical scavenging efficiency on both *σ*^R^ and *σ*_*i*_ parameters, indicating that maximal reactivity occurs when these effects are balanced. Halogen substituents, such as −Cl and −I, exemplify this regime to provide a mild inductive withdrawing character combined with limited resonance donation that facilitates rapid HAT, concurrent to promoting delocalization of spin density across the macrocyclic framework. In contrast, strongly withdrawing groups suppress reactivity by reducing electron density at the reactive β-C–H position, while strongly donating substituents disrupt optimal delocalization through excessive localization or secondary hydrogen-bonding effects.

The PyN_3_ series lacks this additional aryl framework and therefore confines radical stabilization primarily to a single pyridine–amine environment. As a result, substituent resonance effects exert more direct control over the stability of the β-centered radical, leading to a linear dependence of scavenging efficiency on electronic parameters. In contrast, the bis-aryl Py_2_N_2_ scaffold distributes electronic perturbations more broadly across the macrocycle, reducing the dominance of any single substituent effect and giving rise to the observed parabolic behavior.

In principle, alternative hydrogen-atom donor sites may be accessible within the ligand framework. For the hydroxy-substituted derivative, direct H-atom donation from the OH group is chemically plausible. However, the overall substituent-dependent trends observed across the ligand series are not consistent with a dominant OH-driven pathway or consistent with reports of radicals attacking pyridol units [68], this congener shows a strong preference for proton loss and keto–enol tautomerization [51, 58]. Instead, for the radical scavenging process, the strong electronic correlations support formation of a delocalized carbon-centered radical at the β-C–H position adjacent to the pyridine, with the OH group acting primarily as a secondary modulator of reactivity through hydrogen bonding and electronic effects.

Overall, these results establish a unified mechanistic picture in which macrocyclic topology enables ligand-centered HAT and access to a captodative-like electronic regime. The superior radical scavenging performance of Py_2_N_2_ relative to PyN_3_ arises not from differences in intrinsic bond strength, but from the ability of the Py_2_N_2_ scaffold to stabilize transient radical intermediates through cooperative electronic effects. This work highlights a design principle for metal-free macrocyclic antioxidants in which controlled electronic balance within the ligand framework governs radical reactivity.

## 3 | Conclusions

This study demonstrates that pyridine-modified tetraaza macrocycles act as efficient metal-free radical scavengers, with their activity governed by substituent electronics and macrocyclic topology. Both the *para*-substituted Py_2_N_2_ and PyN_3_ families quench the persistent DPPH• radical, but the nature of their electronic control differs fundamentally.

The Py_2_N_2_ scaffold displays rapid and nearly quantitative radical quenching that follows a parabolic dependence on inductive (*σ*_*i*_) and resonance (*σ*^R^) parameters. This revealed an optimal electronic environment where moderate +R and −I effects cooperate to lower the X–H BDE and stabilize the resulting radical. In contrast, the PyN_3_ series shows a linear donor-driven relationship, with activity increasing monotonically with resonance donation. Together, these trends establish that two-pyridine connectivity enables a captodative-like balance of electronic effects inaccessible to single-pyridine systems.

^1^H NMR titrations of I-Py_2_N_2_ with the DPPH• radical corroborate this mechanism, showing ratio-dependent broadening and recovery consistent with preassociation and fast HAT rather than stepwise electron transfer. The transient ligand-centered radical is stabilized through delocalization across both pyridyl rings and the secondary amine bridge, accounting for the high efficiency and reversible reactivity of the Py_2_N_2_ series.

Overall, the results establish a clear structure–reactivity relationship for metal-free macrocyclic antioxidants. Resonance donation accelerates HAT, inductive withdrawal tunes radical stability, and their interplay defines a captodative optimum. These insights provide a predictive framework for designing next-generation redox-active macrocycles whose activity can be precisely modulated by substituent choice, with potential applications in small-molecule antioxidant design, catalytic radical control, and biomimetic redox chemistry.

## Supplementary Material

SI

Supporting Information

Additional supporting information can be found online in the Supporting Information section. Supporting Information is available and includes experimental procedures (general methods, NMR spectroscopy, DPPH assay, and DFT calculations), compound characterization data, and crystallographic information. Additional references associated with the Supporting Information are provided in the main reference list [50–52, 69]. Crystallographic data for ^COOH^PyN_3_ has been deposited at the CCDC under 2522303, and can be obtained from https://www.ccdc.cam.ac.uk/structures.

## Figures and Tables

**TABLE 1 |:** Calculated homolytic C—H BDEs for substituted Py_2_N_2_ compounds in water.

Parent molecule	Path A		Path B	
	Δ*G*, kcal/mol Gas phase	Δ*G*, kcal/mol SMD water	Δ*G*, kcal/mol Gas phase	Δ*G*, kcal/mol SMD water

^H^Py_2_N_2_	103.1	105.3	70.2	76.7
^I^Py_2_N_2_	103.4	105.7	69.8	76.3
^Cl^Py_2_N_2_	104.9	107.4	70.2	76.5
^OH^Py_2_N_2_	104.1	107.3	70.9	76.9
^OCH^_3_Py_2_N_2_	102.8	105.7	70.9	77.6
^CF^_3_Py_2_N_2_	104.5	106.8	68.8	75.7

**TABLE 2 |:** ΔBDE for radical products in gas and water.

Parent molecule	Path A		Path B	
	ΔBDE, kcal/mol Gas phase	ΔBDE, kcal/mol SMD water	ΔBDE, kcal/mol Gas phase	ΔBDE, kcal/mol SMD water

^H^PyN_3_	128.7	105.7	74.9	78.1
^NMe^_2_PyN_3_	102.5	104.4	76.1	86.3
^OH^PyN_3_	104.5	107.5	75.3	85.8
^OMe^PyN_3_	103.3	107.5	75.8	80.1
^I^PyN_3_	103.7	105.9	74.6	85.0
^Cl^PyN_3_	105.2	107.6	74.6	114.1
^CF^_3_PyN_3_	105.1	107.2	74.3	90.2
^CN^PyN_3_	105.3	107.9	74.0	77.2

## Data Availability

Full experimental details are available in the supporting information (.pdf) document. DFT output files are available in https://github.com/kaylagreen-tcu/DPPH_pyridinophanes.
