# Supplementary material for: Metal-Free Ligand-Centered Radical Scavenging in Pyridine-Modified Tetraaza Macrocycles
Source: European J Org Chem. Author manuscript; Available in PMC 2026 Sep 22. (PMC13593359; doi:10.1002/ejoc.70635)
Supplement: Supplementary file 1 [file NIHMS2203784-supplement-SI.pdf]

Metal-Free Ligand-Centered Radical Scavenging in Pyridine-Modified Tetraaza Macrocycles

Christina Mantsorov[a], Hannah K. Pyle[a], Tahmina Afroz[a], Caden-Jack N. Walls[a], Nora Del Bosque[a], Timothy M. Schwartz[a], Chelsea N. Miller[a], Kristof Pota[a], Benjamin G. Janesko[a], Kayla N. Green[a,*]

[a] Department of Chemistry and Biochemistry, Texas Christian University, Fort Worth, TX, USA

* Corresponding Author: kayla.green@tcu.edu

Contents

Equation S1: DPPH• assay calculation for percent radical quenched........ 2

Table S1: Concentration-dependent DPPH• quenching by the para-substituted Py_2N_2 ligands. Values are reported as mean ± SD (n=3) based on absorbance at 517 nm relative to the DPPH• control....... 4

Table S2: Concentration-dependent DPPH• quenching by the para-substituted PyN_3 ligands. Values are reported as mean ± SD (n=3) based on absorbance at 517 nm relative to the DPPH• control....... 4

Table S3: Swain-Lupton induction (σ_i) and resonance (σ^R) parameters for the para substituents and corresponding percent DPPH• quenching at 500 µM for the Py_2N_2 and PyN_3 series........ 5

Figure S1: ^{1}H NMR spectrum of I-Py_2N_2 in DMSO-d_6........ 6

Figure S2: ^{1}H NMR spectrum of DPPH• in DMSO-d_6........ 7

Figure S3: ^{1}H NMR overlaid spectra of 0.5:1 ligand:DPPH• before (red) and after (blue) the addition of 16 µL of D_2O in DMSO-d_6........ 8

Figure S4: ^{1}H NMR overlaid spectra of 1:1 ligand:DPPH• before (red) and after (blue) the addition of 16 µL of D_2O in DMSO-d_6........ 9

Figure S5: ^{1}H NMR overlaid spectra of 2:1 ligand:DPPH• before (red) and after (blue) the addition of 16 µL of D_2O in DMSO-d_6........ 10

Figure S6: ^{1}H NMR overlaid spectra of ligand:DPPH• ratios 2:1 (blue), 1:1 (red), 0.5:1 (green) after the addition of 16 µL of D_2O in DMSO-d_6........ 11

Table S4: Computed, ΔG_A(BDE) = G_A (X-Py_2N_2 radical) + G (Hydrogen radical) - G (X-Py_2N_2)........ 11

Table S5: Computed, ΔG_B(BDE) = G_B (X-Py_2N_2 radical) + G (Hydrogen radical) - G (X-Py_2N_2)........ 11

Table S6: Crystal data, intensity collection, and structure refinement parameters for $^{COOH}PyN_3$........ 12

Table S7: Bond lengths for $^{COOH}PyN_3$........ 13

Table S8: Bond angles for $^{COOH}PyN_3$........ 14

General Methods

All reagents were purchased and used as received. Analytical and anhydrous solvents were purchased from Sigma-Aldrich and ThermoFisher Scientific and were laboratory grade. Water was purified by the 'Millipore' system, with a conductivity of 0.055 μScm^{-1}. All ligands were synthesized according to previously published conditions.[1-3]

NMR Spectroscopy

^1H and ^{13}C NMR were recorded in the stated deuterated solvent on a 400 MHz Bruker AVANCE spectrometer (^1H NMR at 399.782 MHz, ^{13}C at 100.525 MHz) at 298K. Chemical shifts are expressed in ppm and are adjusted to the chemical shift of the residual NMR solvent resonances ($CDCl_3$: ^1H NMR δ = 7.26 ppm, ^{13}C NMR δ = 77.16 ppm, DMSO: ^1H NMR δ = 2.50 ppm, ^{13}C NMR δ = 39.52 ppm, D_2O: ^1H NMR δ = 4.79 ppm). The coupling constants are expressed in Hz.

DPPH• Radical Scavenging Assay

Radical scavenging activity was evaluated using the stable free radical 2,2-diphenyl-1-picrylhydrazyl (DPPH•) in methanol. A stock solution of DPPH• (150 µM) was prepared by dissolving powdered DPPH• in reagent-grade methanol (MeOH). Ligand stock solutions were prepared in MeOH at twice the desired final concentration. Ligands initially obtained as titrated solutions were dried under vacuum prior to dissolution to remove residual water. All solutions were prepared immediately before use, and DPPH• was handled under minimized light exposure to prevent photodegradation.

Assays were conducted in clear, flat-bottom 96-well plates. Blank wells contained 200 µL of MeOH only. Negative control (NC) wells contained 100 µL of MeOH and 100 µL of the DPPH• stock solution. Ligand samples were prepared by adding 100 µL of ligand solution at the appropriate concentration to each well, followed by 100 µL of the DPPH• stock solution, giving a total volume of 200 µL per well. Each concentration was measured in triplicate. The outermost row and column of the plate were not used to avoid edge effects.

Following addition of DPPH•, the plate was immediately covered with aluminum foil and incubated at 21 °C for 30 min. Absorbance measurements were then recorded at λ = 520 nm,[4] using a BioTek µQuant plate reader, with data acquisition performed using Gen5 software (v2.05). Visual documentation of the plate was recorded following measurement.

Percent radical scavenging was calculated relative to the negative control using the average absorbance values of the triplicate wells according to Equation 1.

$$\frac{(\text{Avg. NC Absorbance} - \text{Avg. [Ligand]Absrobance})}{\text{Avg. NC Absorbance}} \text{ x } 100$$

Equation S1: DPPH• assay calculation for percent radical quenched.

NMR Studies of Ligand-Radical Interactions

^1H NMR experiments were performed to probe the interaction between I-Py_2N_2 and the stable radical DPPH• under conditions matching the radical-scavenging assays. All samples were prepared in DMSO-d_6 using freshly prepared stock solutions.

A stock solution of I-Py_2N_2 was prepared in DMSO-d_6, and a separate stock solution of DPPH• was prepared in DMSO-d_6 to allow independent characterization of each component. Control ^1H NMR spectra of the ligand and DPPH• alone were acquired prior to mixing experiments to establish baseline chemical shifts and line shapes.

Mixtures of I-Py_2N_2 and DPPH• were prepared directly in NMR tubes to give three ligand-to-radical ratios while maintaining a constant DPPH• concentration of 13.7 mM. Free ligand concentrations were 26.6 mM (2:1 ligand:DPPH•). 13.3 mM (1:1 ligand:DPPH•), and 6.65 mM (0.5:1 ligand:DPPH•).

All samples were mixed thoroughly by gentle inversion and allowed to stand under ambient laboratory lighting for 15 minutes prior to data acquisition to ensure sufficient interaction. ^1H NMR spectra were then acquired at 298 K. Immediately following acquisition of each spectrum, 16 µL of D_2O was added directly to the NMR tube, the sample mixed again by gentle inversion, and a second ^1H NMR spectrum was recorded. These post-exchange spectra were used to assess the behavior of exchangeable protons and to monitor changes in chemical shift, signal intensity, and line broadening associated with hydrogen-atom transfer and solvent exchange processes.

DFT Modelling

All molecules were modeled from a pre-existing CIF file containing the crystal data of (CCSD: 2522303). Geometry structure optimizations were carried out using Gaussian 16 software package using MO6-2X/def2-TZVP level of theory. Solvent effects were modeled using the SMD implicit water model, with gas phase calculations also performed for comparison. Frequency analysis was performed to ensure that each molecule reached an energy minimum, and spin contamination was checked. For each compound Gibb's free energy was calculated for two possible hydrogen-donor sites: position 3 of the pyridine ring, and beta position of macrocycle adjacent to the pyridine ring. The Gibb's free energy was then used to calculate the bond dissociation energy by the following equation: $BDE = [(parent\ radical + hydrogen\ radical) - (neutral\ molecule)]$.

Table S1: Concentration-dependent DPPH• quenching by the para-substituted Py_2N_2 ligands. Values are reported as mean ± SD (n=3) based on absorbance at 517 nm relative to the DPPH• control.

[Ligand] (μM)	$^{H}Py_2N_2$ (%)	$^{OH}Py_2N_2$ (%)	$^{OMe}Py_2N_2$ (%)	$^{I}Py_2N_2$ (%)	$^{Cl}Py_2N_2$ (%)	$^{CF3}Py_2N_2$ (%)
50	16.0 ± 4.8	-3.1 ± 4.9	21.4 ± 8.2	66.5 ± 1.4	19.0 ± 2.2	3.1 ± 0.2
100	34.1 ± 1.4	8.7 ± 3.2	36.4 ± 3.9	82.1 ± 3.5	35.5 ± 3.6	5.1 ± 0.3
250	62.0 ± 1.9	19.9 ± 6.6	68.9 ± 14.9	90.5 ± 1.8	71.9 ± 6.6	5.6 ± 0.3
500	81.2 ± 7.3	30.4 ± 1.6	84.5 ± 1.2	93.4 ± 0.0	84.5 ± 2.2	10.5 ± 0.3
1000	89.3 ± 1.8	48.9 ± 10.8	91.0 ± 3.6	92.0 ± 2.2	91.2 ± 2.2	16.5 ± 0.2
1500	92.0 ± 2.4	67.9 ± 8.6	92.6 ± 4.3	88.3 ± 4.4	92.5 ± 2.6	25.7 ± 1.3
1750	92.3 ± 2.5	69.8 ± 3.2	93.2 ± 4.8	85.2 ± 6.2	93.2 ± 2.8	ND
2000	93.1 ± 2.8	77.6 ± 0.7	93.5 ± 2.8	81.9 ± 5.1	93.8 ± 6.3	33.5 ± 3.9

*ND denotes no data available

Table S2: Concentration-dependent DPPH• quenching by the para-substituted PyN_3 ligands. Values are reported as mean ± SD (n=3) based on absorbance at 517 nm relative to the DPPH• control.

[Ligand] (μM)	$^{H}PyN_3$ (%)	$^{NMe2}Py_2N_2$ (%)	$^{OH}Py_2N_2$ (%)	$^{OMe}Py_2N_2$ (%)	$^{I}Py_2N_2$ (%)	$^{Cl}Py_2N_2$ (%)	$^{CF3}Py_2N_2$ (%)	$^{CN}Py_2N_2$ (%)
50	9.4 ± 1.0	7.5 ± 6.1	0.4 ± 2.0	0.5 ± 0.0	-0.3 ± 0.2	0.2 ± 0.6	-1.3 ± 3.0	5.0 ± 0.6
100	7.1 ± 2.5	12.5 ± 0.3	-0.4 ± 1.4	3.1 ± 1.6	5.6 ± 5.2	0.9 ± 0.6	1.9 ± 1.3	5.8 ± 0.9
250	4.1 ± 0.6	29.2 ± 0.4	16.4 ± 11.6	10.8 ± 0.8	10.7 ± 0.0	7.7 ± 0.3	9.5 ± 1.0	11.0 ± 0.7
500	6.9 ± 1.0	53.0 ± 0.9	42.8 ± 0.7	30.2 ± 0.5	27.3 ± 0.8	26.1 ± 4.5	27.7 ± 0.6	21.9 ± 0.2
1000	12.3 ± 2.2	72.4 ± 0.6	67.0 ± 0.7	52.6 ± 1.3	49.5 ± 0.7	48.6 ± 3.4	53.6 ± 2.3	39.9 ± 1.0
1500	10.1 ± 0.9	81.2 ± 3.8	77.4 ± 3.9	70.0 ± 3.0	62.6 ± 2.4	62.5 ± 1.8	64.1 ± 0.8	53.1 ± 0.4
1750	9.0 ± 1.1	83.2 ± 1.0	80.4 ± 1.4	73.7 ± 2.2	69.2 ± 0.5	66.0 ± 0.9	66.4 ± 1.5	57.3 ± 1.2
2000	13.2 ± 4.0	84.7 ± 1.1	81.8 ± 1.5	77.5 ± 3.9	72.2 ± 1.8	70.9 ± 1.6	69.8 ± 1.4	63.7 ± 3.0

Table S3: Swain-Lupton induction (σ_i) and resonance (σ^R) parameters for the para substituents and corresponding percent DPPH• quenching at 500 μM for the Py_2N_2 and PyN_3 series.

	Induction Value (σ_i)	Resonance Value (σ^R)	% Radical Quenched (Py_2N_2)	% Radical Quenched (PyN_3)
NMe_2	0.15	-0.98		53.0
OH	0.33	-0.7	30.4	42.8
OMe	0.29	-0.56	84.5	30.2
H	0.03	0	81.2	6.9
I	0.42	-0.24	93.4	27.3
Cl	0.42	-0.19	84.5	26.1
CF_3	0.38	0.16	10.5	21.9
CN	0.51	0.15		27.7

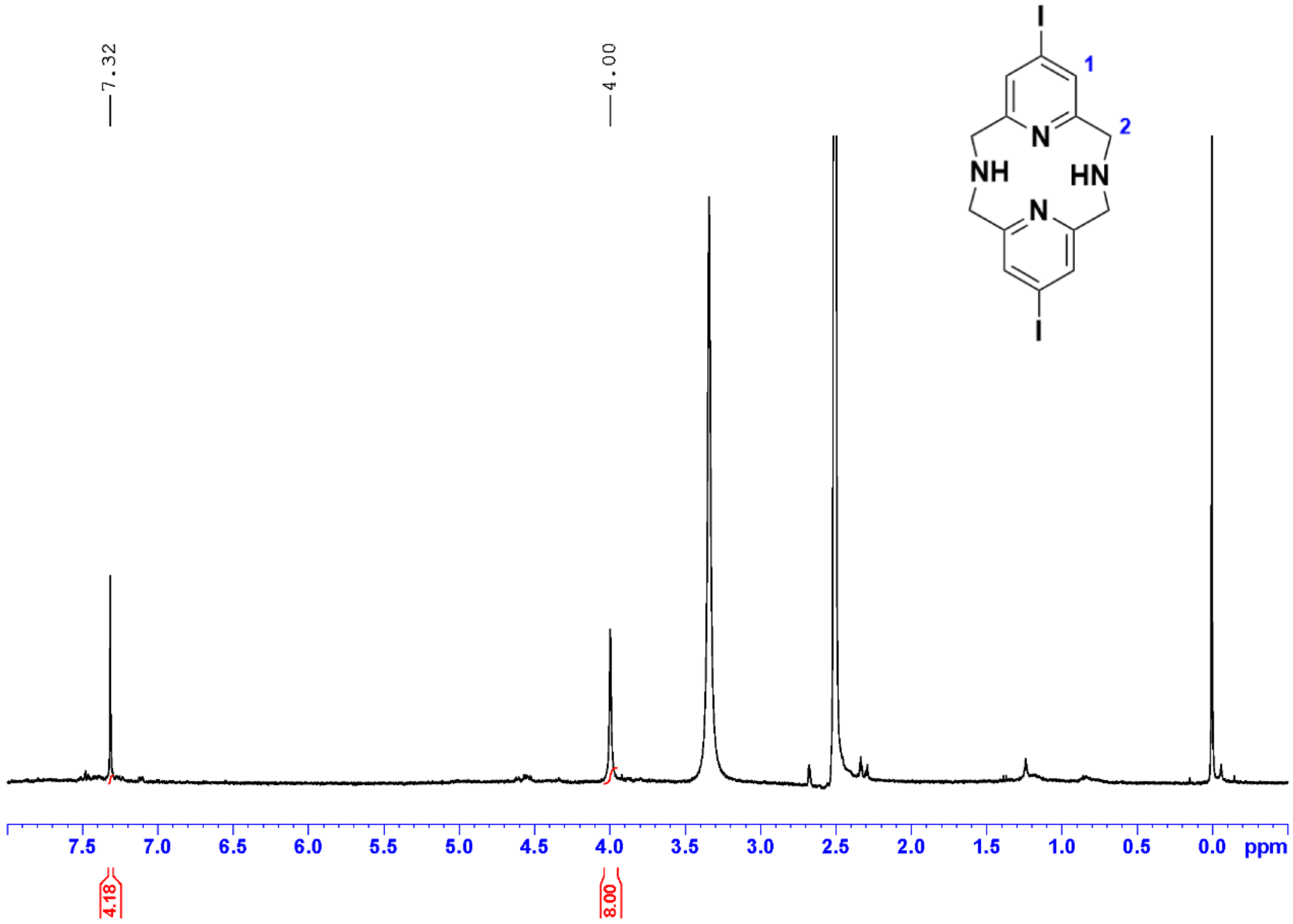

Figure S1: ^{1}H NMR spectrum of I-Py_2N_2 in DMSO-d_6

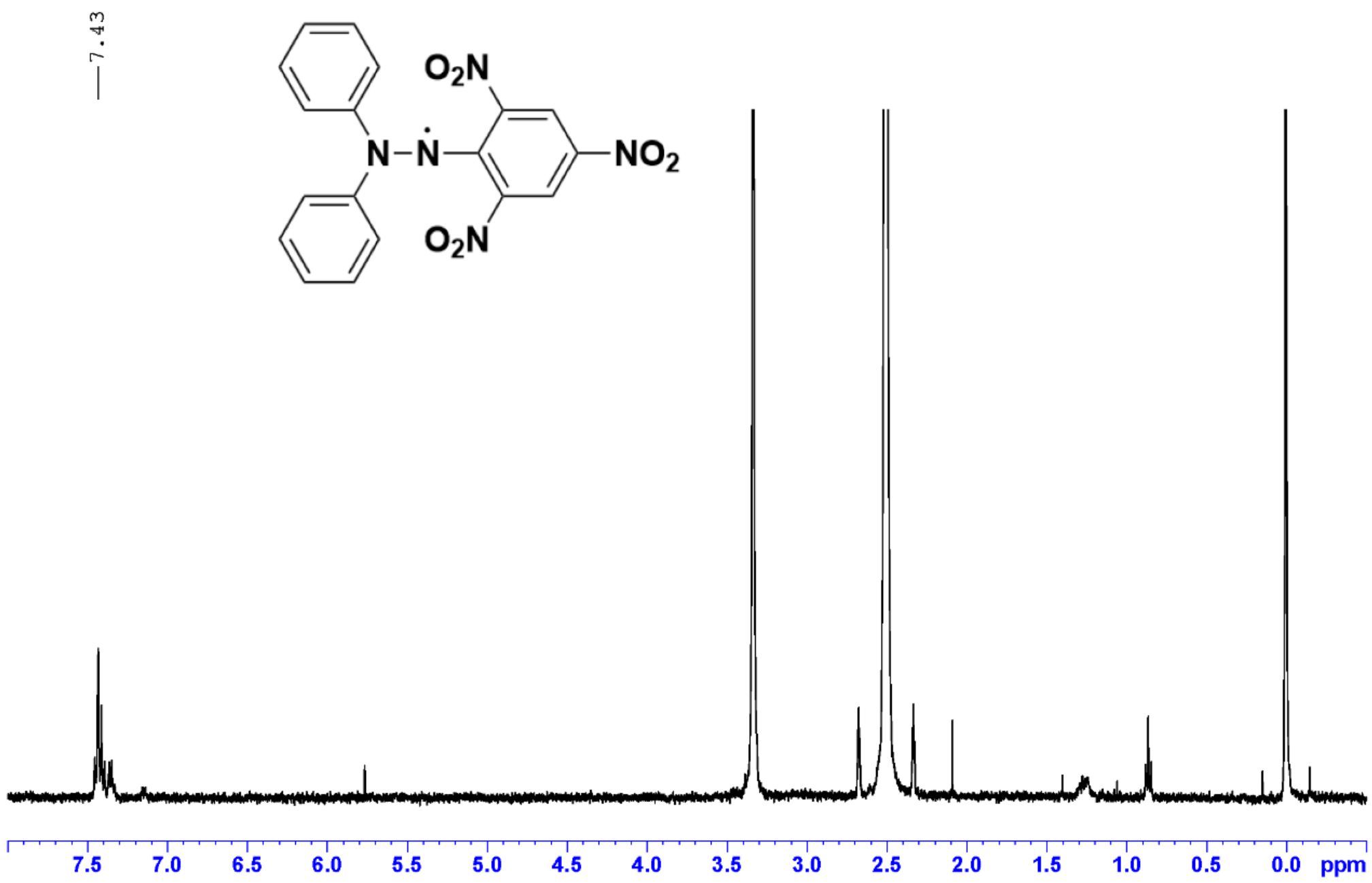

Figure S2: ^{1}H NMR spectrum of DPPH• in DMSO-d_6

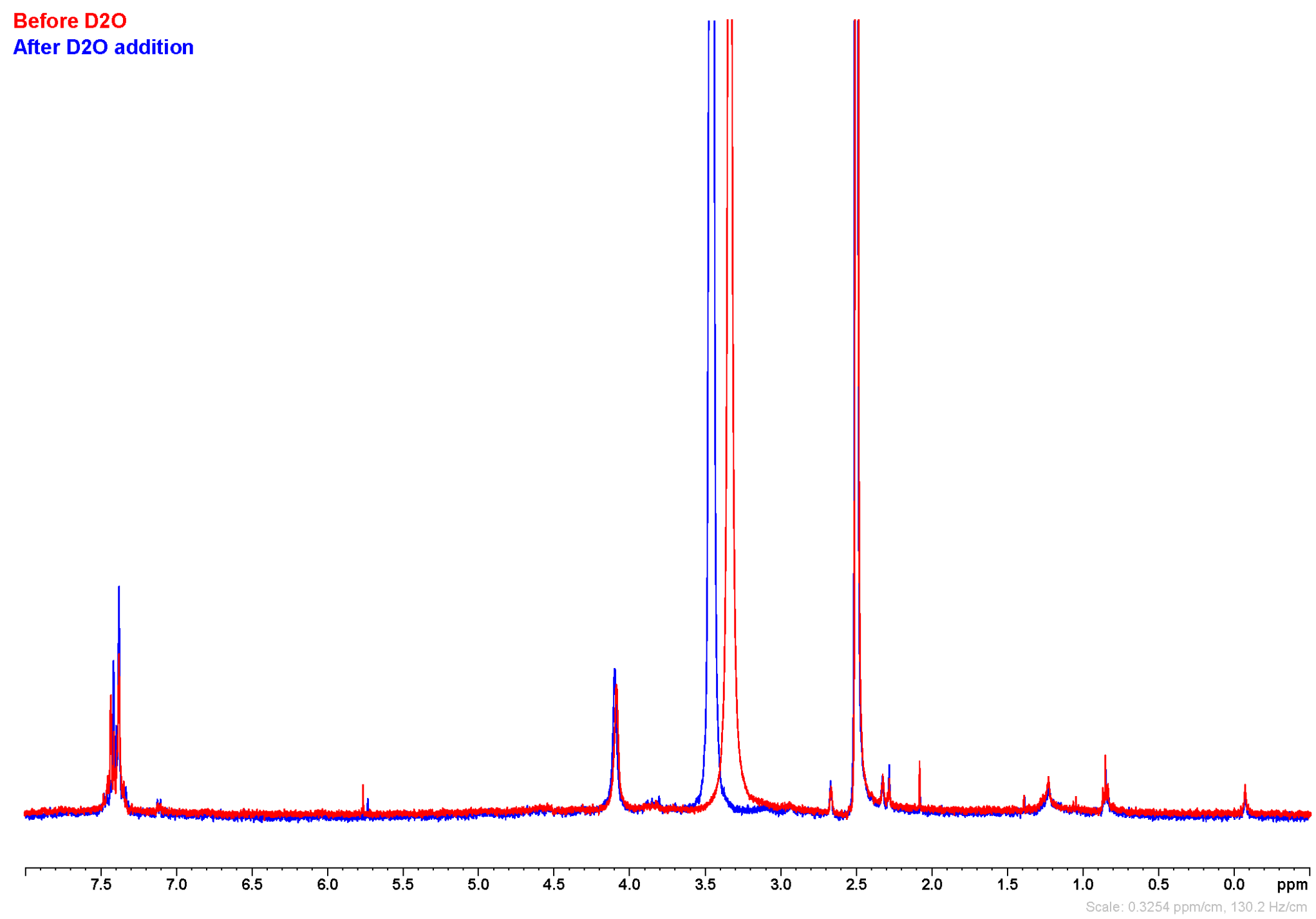

Figure S3: ^1H NMR overlaid spectra of 0.5:1 ligand:DPPH• before (red) and after (blue) the addition of 16 μL of D_2O in DMSO-d_6.

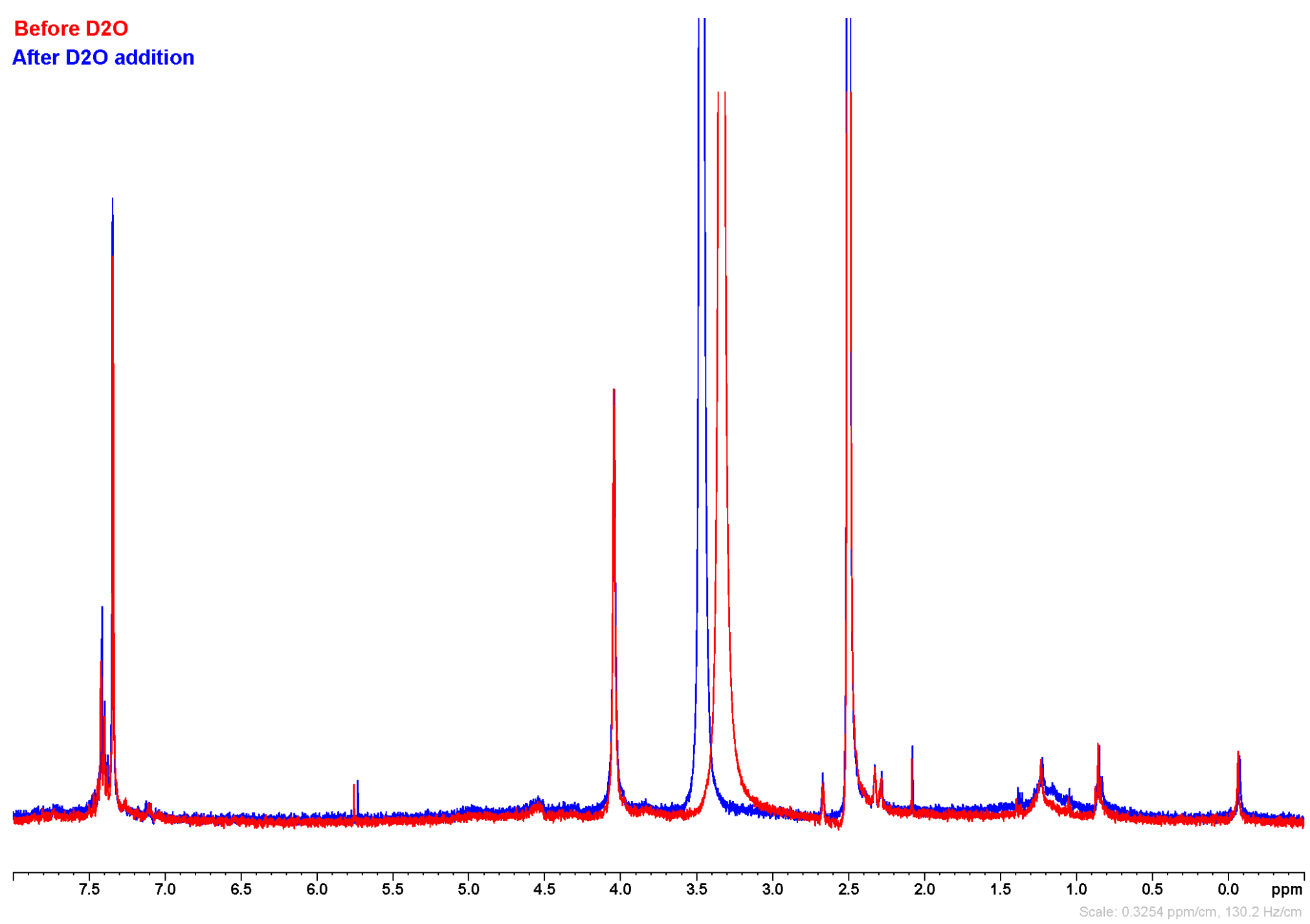

Figure S4: ^{1}H NMR overlaid spectra of 1:1 ligand:DPPH• before (red) and after (blue) the addition of 16 µL of D_2O in DMSO-d_6.

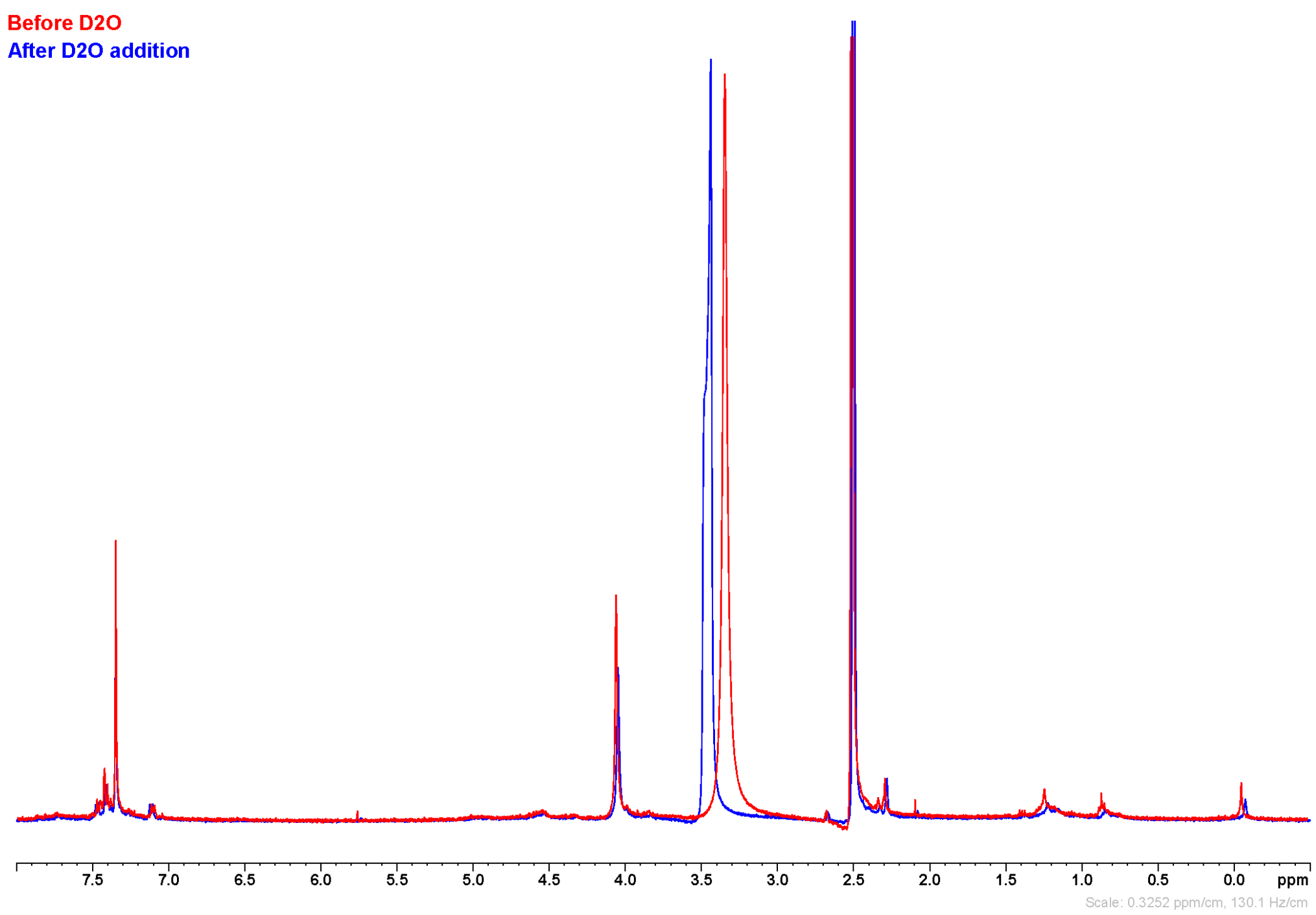

Figure S5: ^{1}H NMR overlaid spectra of 2:1 ligand:DPPH• before (red) and after (blue) the addition of 16 µL of D_2O in DMSO-d_6.

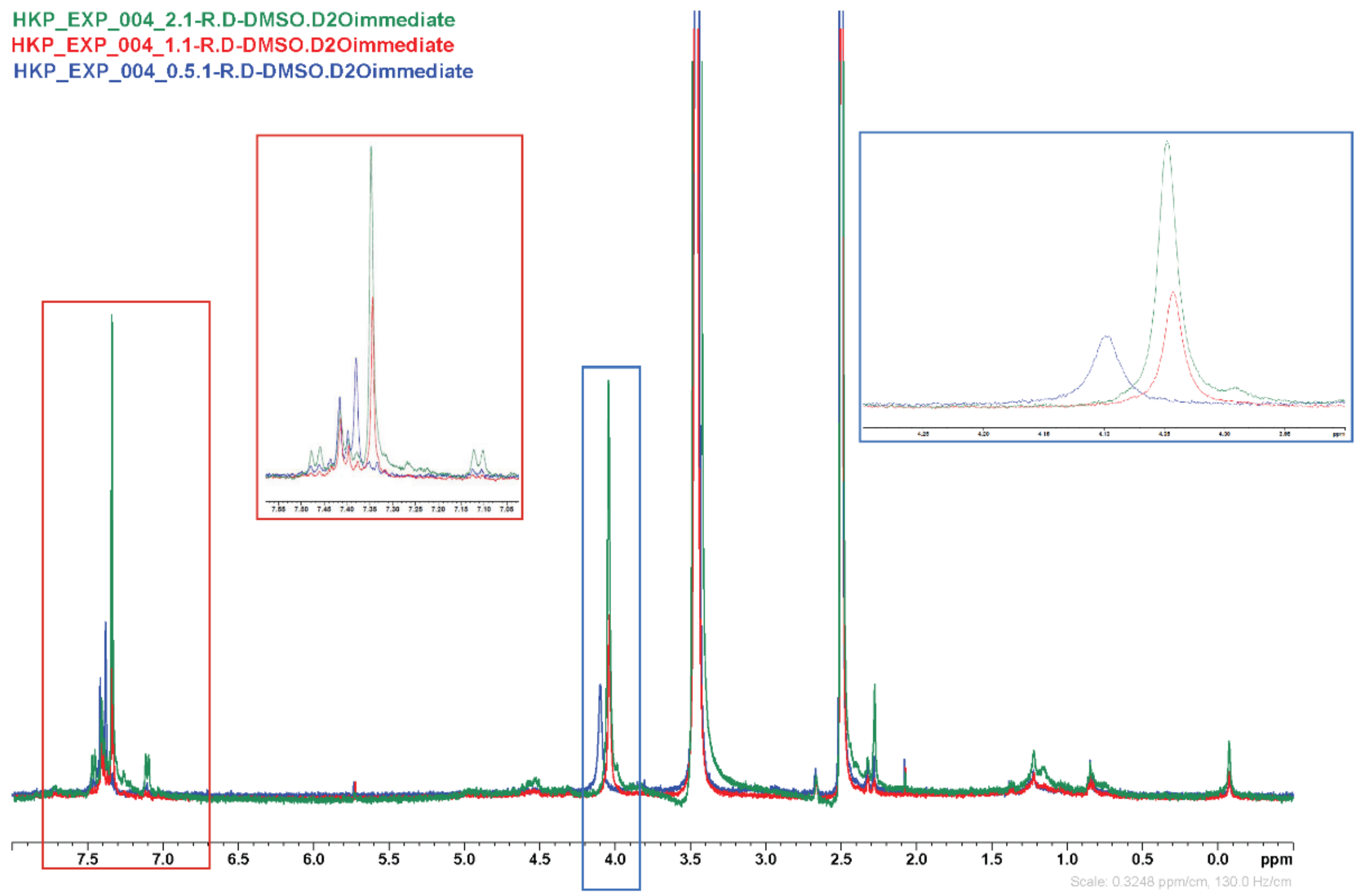

Figure S6: ^{1}H NMR overlaid spectra of ligand:DPPH• ratios 2:1 (blue), 1:1 (red), 0.5:1 (green) after the addition of 16 µL of D_2O in DMSO-d_6.

Table S4: Computed, ΔG_A(BDE) = G_A (X-Py_2N_2 radical) + G (Hydrogen radical) - G (X-Py_2N_2)

SMD	G (Neutral)	G (Radical A_x)	G (Radical H)	ΔG (Hartree)	ΔG_A (Kcal/mol)
H_2O	-1355.944	-1355.269	-0.507	0.168	105.66
MeOH	-1355.942	-1355.267	-0.509	0.167	104.49
DMSO	-1355.936	-1355.261	-0.508	0.167	104.76

Table S5: Computed, ΔG_B(BDE) = G_B (X-Py_2N_2 radical) + G (Hydrogen radical) - G (X-Py_2N_2)

SMD	G (Neutral)	G (Radical B_x)	G (Radical H)	ΔG (Hartree)	ΔG_B (Kcal/mol)
H_2O	-1355.944	-1355.316	-0.507	0.122	76.32
MeOH	-1355.942	-1355.317	-0.509	0.116	73.09
DMSO	-1355.936	-1355.312	-0.508	0.116	72.57

Table S6: Crystal data, intensity collection, and structure refinement parameters for $^{COOH}PyN_3$.

	^{COOH}**PyN**$_3$
CCDC Number	2522303
Empirical formula	$C_{12}H_{24}Cl_2N_4O_{12}$
Formula weight	487.25
Temperature/K	100.(2)
Crystal system	monoclinic
Space group	P21/c
a/Å	8.2154(5)
b/Å	15.8373(9)
c/Å	15.0008(7)
α/°	90
β/°	94.614(2)
γ/°	90
Volume/Å	3 1945.43(19)
Z	4
ρcalcg/cm3	1.664
μ/mm-1	0.407
F(000)	1016
Crystal size/mm3	0.09 × 0.05 × 0.01
Radiation	Mo Kα (λ = 0.71073)
2Θ range for data collection/°	3.74 to 52.74
Index ranges	-10 ≤ h ≤ 10, -19 ≤ k ≤ 19, -18 ≤ l ≤ 17

Table S7: Bond lengths for $^{COOH}PyN_3$.

**$^{COOH}PyN_3$**		
**Atom**	**Atom**	**Length/Å**
O1	C12	1.213(3)
O2	C12	1.323(3)
N1	C1	1.331(3)
N1	C5	1.347(3)
N2	C6	1.493(3)
N2	C7	1.503(3)
N3	C10	1.499(3)
N3	C11	1.488(3)
N4	C8	1.503(3)
N4	C9	1.504(3)
C1	C2	1.396(4)
C1	C6	1.507(3)
C2	C3	1.386(4)
C3	C4	1.396(4)
C3	C12	1.495(4)
C4	C5	1.377(4)
C5	C11	1.514(4)
C7	C8	1.517(4)
C9	C10	1.512(4)
Cl1_1	O1_1	1.468(2)
Cl1_1	O2_1	1.465(2)
Cl1_1	O3_1	1.478(2)
Cl1_1	O4_1	1.4891(19)
Cl1_2	O1_2	1.570(2)
Cl1_2	O2_2	1.447(2)
Cl1_2	O3_2	1.443(2)
Cl1_2	O4_2	1.447(2)

Table S8: Bond angles for $^{COOH}PyN_3$.

$^{COOH}PyN_3$							
**Atom**	**Atom**	**Atom**	**Angle/°**	**Atom**	**Atom**	**Atom**	**Angle/°**
C1	N1	C5	118.0(2)	N4	C9	C10	114.6(2)
C6	N2	C7	116.5(2)	N3	C10	C9	112.5(2)
C11	N3	C10	113.0(2)	N3	C11	C5	109.7(2)
C8	N4	C9	116.2(2)	O1	C12	O2	124.0(2)
N1	C1	C2	122.9(2)	O1	C12	C3	122.2(2)
N1	C1	C6	117.5(2)	O2	C12	C3	113.8(2)
C2	C1	C6	119.6(2)	O1_1	Cl1_1	O3_1	110.29(12)
C3	C2	C1	118.2(2)	O1_1	Cl1_1	O4_1	110.17(12)
C2	C3	C4	119.3(2)	O2_1	Cl1_1	O1_1	109.52(12)
C2	C3	C12	122.3(2)	O2_1	Cl1_1	O3_1	110.83(12)
C4	C3	C12	118.4(2)	O2_1	Cl1_1	O4_1	108.94(12)
C5	C4	C3	118.1(2)	O3_1	Cl1_1	O4_1	107.04(11)
N1	C5	C4	123.3(2)	O2_2	Cl1_2	O1_2	105.78(12)
N1	C5	C11	115.3(2)	O2_2	Cl1_2	O4_2	112.56(13)
C4	C5	C11	121.4(2)	O3_2	Cl1_2	O1_2	104.24(12)
N2	C6	C1	114.0(2)	O3_2	Cl1_2	O2_2	113.28(12)
N2	C7	C8	115.9(2)	O3_2	Cl1_2	O4_2	113.56(13)
N4	C8	C7	111.0(2)	O4_2	Cl1_2	O1_2	106.48(12

References

(1) Mekhail, M. A.; Smith, K. J.; Freire, D. M.; Pota, K.; Nguyen, N.; Burnett, M. E.; Green, K. N. Increased Efficiency of a Functional SOD Mimic Achieved with Pyridine Modification on a Pyclen-Based Copper(II) Complex. *Inorganic Chemistry* **2023**, *62* (14), 5415–5425. DOI: 10.1021/acs.inorgchem.2c04327.
(2) Johnston, H. M.; Pota, K.; Barnett, M. M.; Kinsinger, O.; Braden, P.; Schwartz, T. M.; Hoffer, E.; Sadagopan, N.; Nguyen, N.; Yu, Y.; et al. Enhancement of the Antioxidant Activity and Neurotherapeutic Features through Pyridol Addition to Tetraazamacrocyclic Molecules. *Inorganic Chemistry* **2019**, *58* (24), 16771–16784. DOI: 10.1021/acs.inorgchem.9b02932.
(3) Smith, K. J.; Schwartz, T. M.; Freire, D. M.; Bowers, C. J.; Dunn, S. K.; Bonnell, J. F.; Mekhail, M. A.; Akkaraju, G.; Green, K. N. Rings of Power: Controlling SOD Mimic Activity by the Addition of Pyridine Rings within the Pyridinophane Scaffold. *Inorg Chem* **2024**, *63* (50), 23544–23553. DOI: 10.1021/acs.inorgchem.4c02776 From NLM Medline.
(4) Cos, P.; Hermans, N.; Calomme, M.; Maes, L.; De Bruyne, T.; Pieters, L.; Vlietinck, A. J.; Berghe, D. V. Comparative study of eight well-known polyphenolic antioxidants. *Journal of Pharmacy and Pharmacology* **2003**, *55* (9), 1291–1297. DOI: 10.1211/0022357021693 (accessed 12/16/2025).
